# An Improved Framework for Estimating Organic Carbon Content of Mangrove Soils Using loss-on-ignition and Coastal Environmental Setting

**DOI:** 10.1007/s13157-023-01698-z

**Published:** 2023-06-22

**Authors:** Joshua L. Breithaupt, Havalend E. Steinmuller, Andre S. Rovai, Kevin M. Engelbert, Joseph M. Smoak, Lisa G. Chambers, Kara R. Radabaugh, Ryan P. Moyer, Amanda Chappel, Derrick R. Vaughn, Thomas S. Bianchi, Robert R. Twilley, Paulo Pagliosa, Miguel Cifuentes-Jara, Danilo Torres

**Affiliations:** 1 Florida State University Coastal & Marine Lab, St Teresa, FL USA; 2 Dauphin Island Sea Lab, Dauphin, AL Island; 3 Stokes School of Marine and Environmental Science, University of South Alabama, Mobile, AL USA; 4 Department of Oceanography and Coastal Sciences, Louisiana State University, Baton Rouge, LA USA; 5 School of Geosciences, University of South Florida, St. Petersburg, USA; 6 Department of Biological Sciences, University of Central Florida, Orlando, FL USA; 7 Florida Fish and Wildlife Conservation Commission, Fish and Wildlife Research Institute, St. Petersburg, FL USA; 8 TerraCarbon LLC, Peoria, IL USA; 9 Department of Environmental Engineering Sciences, University of Florida, Gainesville, FL USA; 10 Dept. of Geological Sciences, University of Florida, Gainesville, FL USA; 11 School of the Environment, Yale University, 195 Prospect St, New Haven, CT 06511 USA; 12 Universidade Federal de Santa Catarina, Florianópolis, 88040-900 SC Brasil; 13 Conservation International, 2011 Crystal Dr., Ste. 600, Arlington, VA USA; 14 CATIE - Centro Agronómico Tropical de Investigación y Enseñanza, 30501 Turrialba, Costa Rica

**Keywords:** soil organic carbon, soil organic matter, blue carbon, loss-on-ignition, mangroves, saltmarsh, seagrass

## Abstract

The use of loss on ignition (LOI) measurements of soil organic matter (SOM) to estimate soil organic carbon (OC) content is a decades-old practice. While there are limitations and uncertainties to this approach, it continues to be necessary for many coastal wetlands researchers and conservation practitioners without access to an elemental analyzer. Multiple measurement, reporting, and verification (MRV) standards recognize the need (and uncertainty) for using this method. However, no framework exists to explain the substantial differences among equations that relate SOM to OC; consequently, equation selection can be a haphazard process leading to widely divergent and inaccurate estimates. To address this lack of clarity, we used a dataset of 1,246 soil samples from 17 mangrove regions in North, Central, and South America, and calculated SOM to OC conversion equations for six unique types of coastal environmental setting. A framework is provided for understanding differences and selecting an equation based on a study region’s SOM content and whether mineral sediments are primarily terrigenous or carbonate in origin. This approach identifies the positive dependence of conversion equation slopes on regional mean SOM content and indicates a distinction between carbonate settings with mean (± 1 S.E.) OC:SOM of 0.47 (0.002) and terrigenous settings with mean OC:SOM of 0.32 (0.018). This framework, focusing on unique coastal environmental settings, is a reminder of the global variability in mangrove soil OC content and encourages continued investigation of broadscale factors that contribute to soil formation and change in blue carbon settings.

**Supplementary Information:**

The online version contains supplementary material available at 10.1007/s13157-023-01698-z.

## Introduction

The blue carbon concept has focused global attention on the stocks of organic carbon (OC) stored in coastal vegetated ecosystems that include mangroves, saltmarshes, and seagrasses (McLeod et al. 2011; Fourqurean et al. 2012; Sanderman et al. 2018; Macreadie et al. 2019; (Alongi 2020a). In addition to high rates of productivity, blue carbon ecosystems are important for global carbon budgets because of post-burial longevity of OC in soils (Bouillon et al. 2008; (Alongi 2020b; Kauffman et al. 2020). Remote sensing can be used to characterize and quantify vegetation types, states, and stocks (McCarthy et al. 2018; Goldberg et al. 2020; Lee et al. 2021), and there are increasingly robust global predictions of the distribution of soil OC stocks ranging from continental to global scales (Atwood et al. 2017; Holmquist et al. 2018; Rovai et al. 2018; Sanderman et al. 2018; Kauffman et al. 2020). However, global-scale estimates rely on local-scale empirical measurements to survey and quantify soil and vegetation OC. Ground-based sampling and analytical methods are relatively simple (Howard et al. 2014), though they are labor intensive and can be cost- and time-prohibitive for research projects that lack adequate funding or availability of an elemental analyzer for directly measuring OC.

The lack of funding or access to an elemental analyzer is a major impediment for many coastal wetland practitioners and researchers. Because of this limitation, multiple soil carbon measurement, reporting, and verification (MRV) protocols allow the use of loss-on-ignition (LOI) accompanied by a conversion equation to estimate soil OC content (Kauffman and Donato 2012; Howard et al. 2014; Emmer et al. 2015; World Bank 2021). Additionally, this estimation approach has been used for a subset of observations included in meta-analyses of global blue carbon (e.g., Atwood et al. 2017; Ouyang and Lee 2020). Although there are limitations and uncertainties associated with this estimation approach that make it less accurate than direct measurement, in many cases it remains a necessary method.

### Loss on Ignition Procedures

Loss-on-ignition is an operational quantification of soil organic matter (SOM) that depends on the equally important steps of drying and combustion to sequentially separate water, SOM, and soil inorganic matter. The method consists of two steps: (1) drying the sample to remove water, and (2) combustion to remove SOM without decomposition of carbonates. Drying temperatures of 60–105˚C have been recommended (Ball 1964; Dean 1974) as ignition of organic matter begins at approximately 200˚C (Dabrio et al. 2004). The 105˚C drying temperature is advantageous because it is capable of removing structural waters in some clays (Schulte and Hopkins 1996). Drying durations also vary, but samples are often weighed multiple times until a constant weight has been achieved and no further water loss occurs. Failing to fully dry a sample would result in attributing water weight to the loss of mass during combustion and lead to overestimation of SOM content. Combustion durations and temperatures have been highly variable in the literature because of the need to remove organic matter without de-watering clays or decomposing carbonates, both of which lead to overestimating SOM. Thermogravimetric analysis indicates that calcium carbonate (CaCO_3_) decomposition takes place between 635 and 865˚C (Halikia et al. 2001), and heating time and CO_2_ partial pressure in the furnace atmosphere exert considerable influences on the rate and extent of decomposition (Galan et al. 2013). Decomposition of dolomite [CaMg(CO_3_)_2_] begins to occur around 700˚C (Valverde et al. 2015). Therefore, combustion temperatures of 550 (or less) should not affect carbonates, and this temperature has been frequently recommended in the literature (Davies 1974; Dean 1974; Bengsston and Enell 1986; Heiri et al. 2001; Jones 2001; Wang et al. 2011; Plater et al. 2015; Ouyang and Lee 2020). There is some disagreement about the length of time for combustion at 550˚C, from 2 up to 16 h used for LOI of mangrove soils (Table 2). A longer time ensures more thorough combustion of recalcitrant SOM.

### Conversion Equations for Calculation OC from SOM

**Fig. 1:**
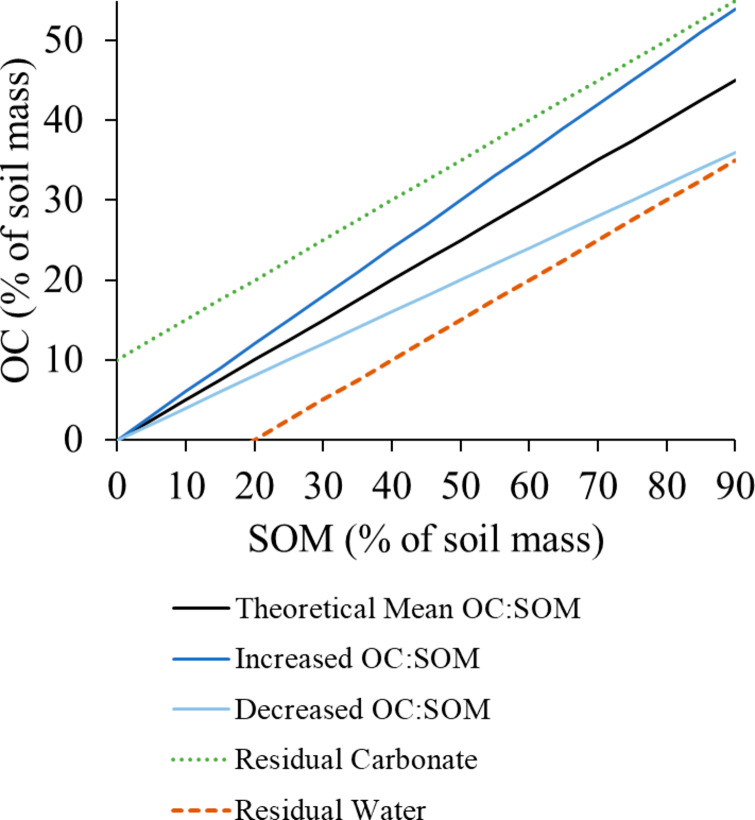
Theoretical linear relationship between organic carbon (OC) and soil organic matter (SOM), taking the form OC = m × SOM + b. The black line represents a relationship where OC:SOM is 0.5. Blue lines represent soils that are enriched or depleted in OC relative to SOM. Dotted and dashed trend lines that intercept the y-axis above or below 0 represent various degrees of procedural error

Figure 1 represents a generalized relationship for which the slope of 0.5 indicates that OC represents 50% of SOM. The equation of this relationship is: OC = m × SOM + b, where m is the slope and b is the y-intercept representing the amount of OC present when SOM equals zero. If the slope increases or decreases, it indicates a relative enrichment or depletion of OC relative to SOM. The green dotted line represents soil for which the y-intercept shows the theoretical presence of OC without any SOM; this is an analysis error which occurs due to the presence of carbonate in elemental analysis samples. The dashed orange line represents soil for which there appears to be zero OC present when SOM is 20% of sediment mass; organic matter compounds are inherently carbon-based, therefore such a situation is interpreted as a methodological error in which the sample was not fully dried and residual water weight is interpreted as SOM mass. Because non-zero intercepts are regarded as error, the b term can be removed and the equation rearranged so that m (slope) = OC/SOM (represented hereafter as OC:SOM).

There are two categories of conversion equations relating SOM to OC in the blue carbon literature. First, general ecosystem equations have been derived from broad spatial-scale datasets for mangroves, saltmarshes, or seagrasses (Table 1). Second, there are region-specific equations derived from empirical datasets (Table 2); in this study we address region-specific equations only for mangroves. Hereafter, we refer to general ecosystem equations for the broad-spatial scale equations in Table 1, and region-specific equations for the equations presented in Table 2 and in our dataset.

**Table 1:** Previously published blue carbon conversion equations for using loss-on-ignition measurements of soil organic matter (SOM) to estimate soil organic carbon (OC) content. Additional equation descriptions include total sample numbers used for each equation (n), number of samples with SOM greater than 60% (n > 60%), SOM range of dataset, ratio of OC:SOM identified by the conversion equation, and the correlation coefficient R^2^). Reference 1 samples were dried overnight at 105 ˚C, then combusted at 450 ˚C for 8 h. References 2 and 3 are meta-analyses of published literature and represent multiple drying and combustion times and temperatures.

Ecosystem	Equation	n	n_> 60%_	SOM Range (%)	OC:SOM	R^2^
Saltmarsh^a^	OC = 0.0025 (± 0.0003) × SOM^2^ + 0.40 (± 0.01) × SOM	250	15	0–73	0.41–0.65	0.99
Saltmarsh^b^	OC = 0.52 (± 0.006) × SOM − 1.17 (± 0.12)	344	6	0–70	0.52	0.99
Seagrass^c^	OC = 0.40 × SOM − 0.21	1667	0	< 20	0.40	0.87
Seagrass^c^	OC = 0.43 × SOM − 0.33	1748	14	0-100	0.43	0.96
Mangrove^b^	OC = 0.21 (± 0.01) × SOM^1.12 (±0.02)^	1534	22	0–80	0.25–0.36	0.86

References: ^a^Craft et al. 1991, ^b^Ouyang and Lee 2020, ^c^Fourqurean et al. 2012

**Table 2:** Region-specific mangrove conversion equations for estimating organic carbon (OC) content from loss-on-ignition (LOI) measurements of soil organic matter (SOM) in mangrove soils: OC = m(SOM) + b. UCW: until constant weight; n/a: not available. Abbreviations in parentheses following regional names are coastal environmental setting (CES) after Worthington et al. (2020): terrigenous delta (TD), terrigenous estuary (TE), terrigenous lagoon (TL), terrigenous open coast (TOC), carbonate estuary (CE), and carbonate open coast (COC); N/A indicates reference includes more than one CES.

Location	n	Drying Procedure	LOI Procedure	SOM Mean; Range (% of dry soil mass)	m	b	R^2^
Mui Ca Mau National Park, Vietnam (TD)^a^	225	60°C, UCW	550°C, 5 h	7.8; 3–20	0.23	0.2	0.56
Chek Jawa, Singapore (TE)^b^	40	60°C, 72 h	500°C, 4 h	14.0; 0–30	0.24	1.2	0.46
Hau Loc, North-Central Vietnam (TOC)^c^	n/a	60°C, UCW	550°C, n/a	7.3; 2–11	0.25	-2.7	0.72
Ceará State, Brazil (TE)^d^	60	n/a	450°C, 2 h	12.7; 5–30	0.27	n/a	0.82
Can Gio Mangrove Forestry Park, Vietnam (TD)^e^	316	60°C, UCW	550°C, 5 h	11.2; 5–20	0.35	-1.3	0.8
São Paulo State, Brazil (TL)^f^	36	60°C, UCW	550°C, 2 h	16.5; 5–27	0.35	-0.69	0.84
Matang, Malaysia (N/A)^g^	103	60°C, n/a	550°C, 4 h	n/a; 20–55	0.37	-0.1	0.61
Dong Rui, Vietnam (TOC)^h^	30	60°C, UCW	550°C, 5 h	7.6; 3–17	0.41	n/a	0.79
Tampa Bay, FL USA (CE)^i^	57	105°C, 24 h	550°C, 3 h	n/a; 2–69	0.42	n/a	0.95
Republic of Palau (COC)^j^	78	105°C, 24 h	400°C, 16 h	n/a; 15–52	0.42	2.9	0.59
Gazi Bay and Vanga, Kenya (N/A)^k^	70	60°C, 24–48 h	550°C, 2 h	n/a	0.43	0.2	0.64
Chonburi Province, Gulf of Thailand (TE)^l^	29	105°C, UCW	550°C, 4 h	11.1; 6–17	0.5	0.10	0.25
Jervis Bay, NSW Australia (TE)^m^	32	60°C, UCW	375°C, 16 h	5.8; 0–16	0.51	0.2	0.95
Muisne Island, Ecuador (N/A)^n^	> 50	105°C, 12 h	500°C, 12 h	17.9; 0–35	0.87	-5.8	0.89

References: ^a^Tue et al. 2014, ^b^Phang, Chou, and Friess 2015, ^c^Van Hieu et al. 2017, ^d^Nóbrega et al. 2015, ^e^Dung et al. 2016, ^f^Rovai et al. 2021, ^g^Adame et al. 2018 Supplementary Material, ^h^Nguyen et al. 2016, ^i^Radabaugh et al. 2018, ^j^Kauffman and Donato 2012, ^k^Gress et al. 2017, ^l^Chaikaew and Chavanich 2017, ^m^Owers et al. 2016, & ^n^Delvecchia et al. 2014

Although Fig. 1 depicts a generalized relationship where OC:SOM equals 0.5, in actuality there is a wide range of equation slopes reported in the literature for terrestrial and marine soils and sediments. General blue carbon ecosystem equations indicate OC:SOM ranges from 0.25 to 0.65 (Table 1). The sample sets for these equations have similar ranges of SOM, but the number of samples with high SOM content (i.e., greater than 60%) is proportionally quite small for most of these equations (Table 1). This suggests these equations may not be well-suited for high SOM soils that can occur in some coastal wetlands. When the coastal blue carbon methods manual was published by Howard et al. (2014), a regional equation from Palau was the only available conversion equation specific to mangroves; the equation had a slope of 0.42, but a relatively weak correlation coefficient of 0.59 (Kauffman and Donato 2012) (Table 2). At least 14 region-specific equations have been developed for mangroves in the past decade, with slope values (i.e., OC:SOM) ranging from 0.23 to 0.87, and correlation coefficients ranging from 0.25 to 0.95 (Table 2). The slopes are similar to findings from a review of conversion equations for non-mangrove settings (Pribyl 2010), that ranged from a low of 0.34 in ponderosa pine forests of northern Arizona, USA (Abella and Zimmer 2007) to a high of 0.73 in eucalypt plantation soils in Tasmania, Australia (Wang et al. 1996). Other mangrove research has relied on conversion equations from non-mangrove, upland soils with a slope of 0.52 (Breithaupt et al. 2012; Adame et al. 2013; Brown et al. 2016) or 0.58 (Eid et al. 2016; Eid and Shaltout 2016). The polynomial saltmarsh model of Craft et al. (1991) (Table 1) has also been applied to mangrove soils (Guerra-Santos et al. 2014; Hong et al. 2017).

### Objectives

A key objective of this study was to provide a framework for understanding the wide variation in slopes reported in these conversion equations (Tables 1 and 2). The methodological errors and systematic biases that can occur during the measurement of LOI and OC have been well-documented and recognized for decades (Mook and Hoskin 1982; Harris et al. 2001; Heiri et al. 2001; Brodie et al. 2011; Howard et al. 2014) (Fig. 1). A range of drying procedures have been used for determining mangrove OC:LOI equations, including variations in drying time from 12 to 24 h or until constant dry weight between measurements was achieved. This could lead to some discrepancies between studies, but these are all common procedures, so their effect is not expected to be large. The times and temperatures of combustion range quite widely, from 375–550˚C and from 2 to 16 h (Table 2). However there does not appear to be a systematic bias leading to higher or lower than expected values because of combustion temperature. For example, the lowest slope of 0.23 was determined using conservative drying and combustion procedures that are well within norms (Tue et al. 2014; Table 2). The highest slope of 0.87 was determined using a similarly conservative drying procedure and combustion temperature; theoretically the 12-hour combustion time would contribute to a higher SOM, which would contribute to a lower OC:SOM rather than the highest slope in the dataset. However, the lack of a standardized approach is an ongoing cause of uncertainty, as demonstrated by the variety of methods employed for deriving mangrove conversion equations (Table 2). Nonetheless, method differences do not explain the variation that occurs when methods are applied consistently and there has been almost no explicit evaluation of environmental factors that contribute to these equation differences.

In their derivation of the general saltmarsh conversion equation, Craft et al. (1991) proposed that variation in OC:SOM was related to soil age. They noted young soils with very low OM content of 1% had an OC:SOM value of 0.40, similar to the OC:OM of the local emergent vegetation. Conversely, they proposed increased OC:SOM values of 0.55–0.60 in more mature soils with up to 60–80% SOM were due to the greater proportion of more recalcitrant organic compounds (Craft et al. 1991). While this explanation equating young soil with low SOM content and mature soils with high SOM content may be useful in ecosystems constrained by the same environmental boundaries, its applicability is limited when making comparisons between systems where OC and SOM may be differentially supplied either autochthonously, as in carbonate systems with limited terrigenous inputs (Woodroffe 1993), or allochthonously, as in systems where sediment supply is dominated by terrigenous sources (Balke and Friess 2016; Jennerjahn 2020).

Global classification frameworks are useful for explaining drivers and variation in macroscale ecological differences among mangrove ecosystems defined by primary drivers of hydrology, geomorphology, and climate (Lugo and Snedaker 1974). Indeed, a classic framework that differentiates global coastlines into conspicuous coastal environmental settings based on mineral sediment type and quantity, geomorphic setting, nutrient availability, and regional climate (Thom 1984; Woodroffe 1993) has empirically been shown to explain variations in mangrove soil traits including OC density (mg cm^− 3^), stocks (Pg), C:N:P stoichiometric ratios (Rovai et al. 2018; Twilley et al. 2018), and aboveground carbon stocks (Rovai et al. 2021b). Recently, these coastal environmental settings have been made available as a high resolution spatially explicit map of mangroves (Worthington et al. 2020). In this map, global mangrove cover area was classified and assigned to one of two broad sedimentary settings based on the dominant source of mineral sediment: in terrigenous settings, sediment is minerogenic and derived from terrestrial sources, and in carbonate settings inorganic sediment is derived from in situ calcareous processes. According to this biophysical typology, coastal environmental settings are further classified as deltas, estuaries, lagoons, and open coasts (Worthington et al. 2020).

**Fig. 2:**
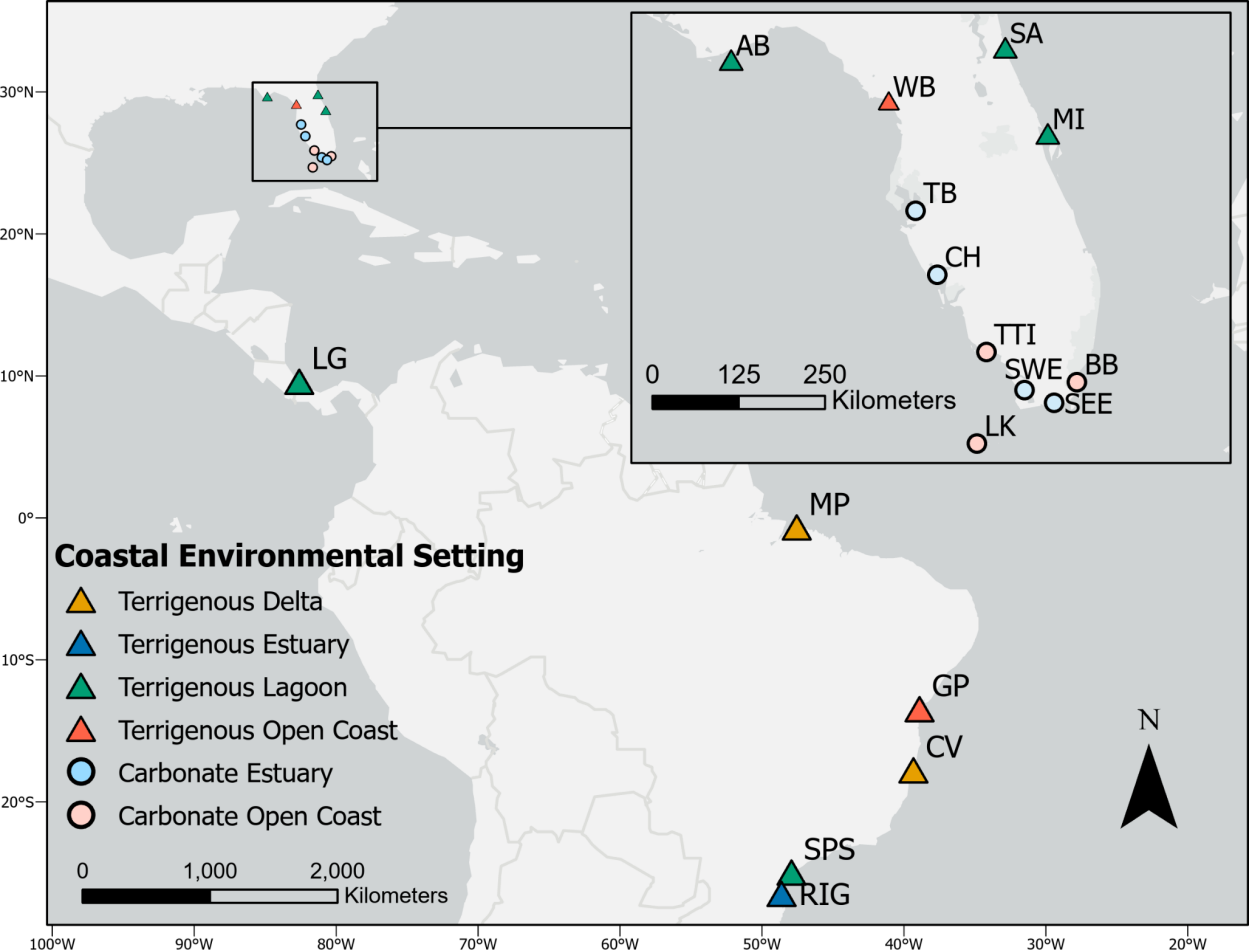
Soil core locations and assigned environmental settings after Worthington et al. (2020) (adapted) in Florida, USA, Costa Rica, and Brazil. From North to South, abbreviations are listed as follows: SA, St. Augustine; AB, Apalachicola Bay; WB, Waccasassa Bay; MI, Merritt Island; TB, Tampa Bay; CH, Charlotte Harbor; TTI, Ten Thousand Islands; SWE, southwest Everglades; BB, Biscayne Bay; SEE, southeast Everglades; LK, Lower Keys; LG, Laguna Gandoca; MP, Marapanim; GP, Garapua; CV, Caravelas; SPS, São Paulo State, and RIG, Ratones, Itapoá, Guaratuba

Here we investigated if this framework could be informative for explaining differences among conversion equations for estimating soil OC content in mangrove ecosystems. Using a primary dataset that spans 6,365 km from 27.45° South to 29.83° North across the tropics in the western hemisphere, including eleven geographic regions in Florida, USA, one region in Costa Rica, and five regions in Brazil (Fig. 2), the objectives of this research were to (1) investigate variability of conversion equations among these regions, (2) evaluate the hypothesis that OC:SOM is positively correlated with SOM content (Craft et al. 1991), (3) evaluate OC:SOM as a function of sedimentary and coastal environmental settings, and (4) recommend steps for users to select the most appropriate conversion equation.

## Materials and Methods

### Study Regions and Typology

The dataset for this study (Supplementary Dataset 1) was compiled from previous research in 17 mangrove regions (Supplementary Table S1; Fig. 2). These regions represent a wide variety of settings that are occupied by mangroves. Regions include the northernmost extent of mangrove range expansion along the Gulf of Mexico and Atlantic Ocean coasts of North America and near the southern extent of range expansion in Brazil (Fig. 2). The diverse site histories provide a comprehensive approach from which to examine mangrove OC:SOM trends across a broad geographical scale in the range of coastal environmental settings where mangroves occur. Readers are referred to Supplementary Table S1 for the original references which provide environmental context for each region.

Each region was designated to a sedimentary and coastal environmental setting (Fig. 2) following the Worthington et al. (2020) typology, except in cases where differences were identified by expert opinion of our authors or information specified in the literature. First, five of our regions were not included in the 2016 Global Mangrove Watch map used by Worthington et al. (2020); these were Merritt Island, St. Augustine, Waccasassa Bay, Apalachicola Bay, and Laguna Gandoca. Worthington et al. (2020) noted that joining their typology with the Global Mangrove Watch map for identifying extent of mangroves was not intended for prediction of mangrove presence or absence. Second, we changed the sedimentary setting for Tampa Bay, Charlotte Harbor, Ten Thousand Islands, and Biscayne Bay from terrigenous to carbonate. Previous work by our group has identified that CaCO_3_ content of Tampa Bay mangroves ranged from 7.4 ± 2.7 to 9.4 ± 6.3% and from 5.8 ± 0.7 to 58.5 ± 0.9 for Southwest Everglades mangroves (Breithaupt et al. 2019). We also include previously unpublished data showing that site average soil CaCO_3_ content was up to 22.2 ± 2.0, 28.5 ± 4.1, and 24.7 ± 8.2 for Charlotte Harbor, Ten Thousand Islands, and Biscayne Bay, respectively (Supplementary Dataset 2). Lastly, we changed the geomorphic designation of the Southwest Everglades and Southeast Everglades regions from lagoon to estuary. While there are locations within each region that could fit the designation of either setting, the sites where our samples were collected most closely resemble the estuarine designation. The same process was followed to assign previously published regional mangrove equations (Table 2) to a coastal environmental setting.

### Sample Collection & Analyses

Soil cores were collected by various methods including gouge auger, half-barrel chamber peat corer, vibra-corer, and polyvinylchloride and polycarbonate push cores (Supplementary Table S1). Cores were sectioned in interval thicknesses ranging from 1 to 5 cm over soil depths up to 1 m. Samples were dried at 60, 70, or 105°C in an oven until constant dry weight was observed or dried in a freeze dryer (Table S1). Although different drying temperatures might lead to discrepancies between SOM estimates, we do not believe these differences influenced our findings because (a) these temperatures have been shown to sufficiently dry samples without risking SOM combustion, and (b) approaches in each lab were careful to ensure constant weight of the dried sample and no residual moisture content. After drying, samples were homogenized using a mortar and pestle or ball mill. Sample organic matter content was determined via LOI following combustion at 550 °C in a muffle furnace for three hours (Radabaugh et al. 2018). Our use of three hours instead of four (Heiri et al. 2001) for mangrove sediments was due to observations of negligible mass loss during the final hour of combustion. The CaCO_3_ content of a subset of these samples (Supplementary Dataset 2) was measured using a sequential loss-on-ignition procedure based on the difference in mass lost between 550 and 990℃ (Dean 1974; Breithaupt et al. 2019). Organic carbon content was determined via elemental analyzer. Measurements of OC were made using (1) a Carlo Erba 1500 CN elemental analyzer for St. Augustine and Waccasassa Bay, (2) an Elementar Vario Micro Cube CHNS Analyzer for Apalachicola Bay, Merritt Island, the Little Manatee River in Tampa Bay, and the Peace River in Charlotte Harbor, and (3) a PDZ Europa ANCA-GL (Automated Nitrogen Carbon Analyzer-Gas Solids Liquids) elemental analyzer for all remaining sites (see Table S1 for citations of the original publications with complete methods). Samples containing carbonate sediment were fumigated with 12 M hydrochloric acid prior to measurement of OC (Harris et al. 2001).

### Data Analysis

Statistical analyses were conducted using IBM® SPSS® Statistics Version 27. All data were compiled from previous publications whose objectives were not the same as this investigation; therefore, we began this study by inspecting the dataset for outliers related to the OC and SOM relationship. Samples with OC:SOM ratios greater than or equal to 1.0 or less than or equal to 0.0 were removed. Values greater than or equal to 1.0 may occur if the inorganic carbon was not completely removed and samples with OC:SOM less than 0.0 occur as a result of sample preparation error. A modified Thompson Tau test was used to identify and remove statistical outliers from each location. Simple linear and polynomial regressions were used to assess the relationship between OC and SOM content for the complete dataset and each region. Comparisons of mean ratios of OC:SOM by sedimentary setting were conducted with independent t tests; differences between geomorphic setting, and coastal environmental setting were conducted via 1-way ANOVA and post-hoc comparisons using Tukey’s Highly Significant Difference with alpha set for 0.05. The purpose of this research was to improve the utility of the LOI process for estimating mangrove soil OC content at the regional scale. Therefore, each of the conversion equations generated in this effort, as well as the most recent global mangrove equation (Ouyang and Lee 2020), were tested on our data at the regional level (Supplementary Table S2). This was done by testing whether the average of the residuals (between observed and modeled OC values) was different from zero using individual t-tests for each conversion equation within each region. For regions whose residuals did not meet the assumptions of normality after transformation (determined using the Shapiro-Wilk test), differences from zero were tested with the non-parametric 2-sided One Sample Wilcoxon Signed Rank Test. All reported uncertainties represent 1 standard error unless stated otherwise.

When comparing OC conversion equations, there is a small but important point to remember regarding the units of OC and SOM used in the regression. In this study, both x and y variables (SOM and OC, respectively) are represented as percentages between 0 and 100, whereas in other studies (Craft et al. 1991) they are represented as decimal fractions between 0 and 1. The calculated value of the slope will be the same regardless of which format is used; however, the intercept term requires adjustment when converting or comparing between formats. For example, when the variables are entered as percentages, an intercept of 10 represents 10% OC and 0.10 represents 0.10%. Conversely, when the variables are decimal fractions, an intercept of 0.10 represents 10% OC and 0.01 represents 1%. All variables were standardized to percentages for statistical analyses.

### Analysis of Conversion Equation Use in Global Stock Estimates

References that were used in global soil OC stock assessments by Atwood et al. (2017) and Ouyang and Lee (2020) were reviewed to identify those that measured OC and those where OC was estimated from LOI (Supplementary Dataset 3). For those that estimated OC, the study locations were spatially joined to the Worthington et al. (2020) map to identify the coastal environmental setting to which each belonged. Conversion equations specific to each setting were then applied (Table 3). Both datasets (Atwood et al. 2017; Ouyang and Lee 2020) included five and eight samples, respectively, that were identified by Worthington et al. (2020) as carbonate lagoons. Because our dataset did not include any regions classified as carbonate lagoon, we used the carbonate open coast conversion equation for these sites. A non-parametric Wilcoxon Signed Rank Test was conducted to test for differences between estimates derived using the coastal environmental setting conversion equations (Table 3) with the estimated OC derived from the two previous studies.

**Table 3:** Mangrove conversion equation values for predicting organic carbon (OC) content of soil organic matter (SOM) by Region, Coastal Environmental Setting (CES), and Sedimentary Setting. Regional and CES equations are in linear form: OC = m (± 1 S.E.) × SOM + b (± 1 S.E.); Sedimentary setting equations are in quadratic form. All equations are significant at the level of p < 0.001. Abbreviations in parentheses following regional names are coastal environmental setting: terrigenous delta (TD), terrigenous estuary (TE), terrigenous lagoon (TL), terrigenous open coast (TOC), carbonate estuary (CE), and carbonate open coast (COC)

Region	Slope	Intercept	R^2^	n
St. Augustine (TL)	0.283 (± 0.012)	− 0.3 (± 0.1)%	0.96	29
Apalachicola Bay (TL)	0.392 (± 0.016)	− 0.1 (± 0.3)%	0.96	27
Waccasassa Bay (TOC)	0.360 (± 0.028)	− 2.8 (± 0.9)%	0.87	28
Merritt Island (TL)	0.460 (± 0.009)	− 0.3 (± 0.3)%	0.98	50
Tampa Bay (CE)	0.427 (± 0.016)	− 0.2 (± 0.6)%	0.9	80
Charlotte Harbor (CE)	0.465 (± 0.015)	1.2 (± 0.7)%	0.96	43
Ten Thousand Islands (COC)	0.442 (± 0.019)	0.9 (± 1.0)%	0.75	183
Biscayne Bay (COC)	0.439 (± 0.032)	+ 1.5 (± 1.5)%	0.88	29
Southwest Everglades (CE)	0.515 (± 0.006)	− 2.2 (± 0.4)%	0.93	543
Southeast Everglades (CE)	0.519 (± 0.020)	-4.5 (± 1.1)%	0.95	36
Lower Keys (COC)	0.521 (± 0.033)	− 1.9 (± 2.3)%	0.92	23
Laguna Gandoca (TL)	0.687 (± 0.036)	-15.5 (± 2.3)%	0.95	21
Marapanim (TD)	0.240 (± 0.038)	0.1 (± 0.5)%	0.80	12
Caravelas (TD)	0.321 (± 0.022)	-2.1 (± 0.5)%	0.86	36
Garapua (TOC)	0.369 (± 0.027)	0.1 (± 0.5)%	0.85	36
Ratones, Itapoa, & Guaratuba (TE)	0.249 (± 0.034)	0.0 (± 0.4)%	0.63	34
São Paulo State (TL)	0.355 (± 0.026)	-0.7 (± 0.5)%	0.85	36
**Coastal Environmental Setting**				
Carbonate Estuary	0.506 (± 0.005)	-1.7 (± 0.3)%	0.94	702
Carbonate Open Coast	0.470 (± 0.014)	-0.6 (± 0.7)%	0.82	235
Terrigenous Delta	0.287 (± 0.019)	-1.2 (± 0.4)%	0.84	48
Terrigenous Estuary	0.250 (± 0.033)	0.9 (± 3.7)%	0.64	34
Terrigenous Lagoon	0.472 (± 0.007)	-1.7 (± 0.2)%	0.97	163
Terrigenous Open Coast	0.281 (± 0.021)	0.7 (± 0.5)%	0.75	64
**Sedimentary Setting**	**Quadratic Equation**		**R** ^2^	**n**
Terrigenous Setting	0.004 (± 0.000) × SOM^2^ + 0.217 (± 0.022) × SOM + 0.3 (± 0.3)%		0.94	309
Carbonate Setting	0.002 (± 0.000) × SOM^2^ + 0.326 (± 0.019) × SOM + 1.8 (± 0.4)%		0.93	937

## Results & Discussion

### Regional Soil Organic Matter Content

The dataset consisted of 1,246 samples from soils less than 1 m deep at 34 sites representing 17 regions (Supplementary Table S1, Supplementary Dataset 1; Fig. 2). The overall mean (± 1 S.E.) SOM content was 44.37 ± 0.68%, with regional means that ranged from 9.9% in the Ratones, Itapoá, and Guaratuba (RIG) sites in southern Brazil to 69.2% in the Lower Keys (LK) sites in Florida (Supplementary Table S1). Overall, out of the 17 regions, ten had mean SOM content of less than 30%, four had mean SOM between 30 and 50%, and three had mean SOM greater than 50% (Supplementary Table S1).

A strength of this dataset is the wide range of SOM content (1.1–87.1%), including more than 30% of samples having greater than 60% SOM. This contrasts with general blue carbon ecosystem conversion equations for which the datasets contained fewer than 6% of samples with more than 60% SOM content (Table 1). Of the published regional mangrove conversion equations, only three of the fourteen had samples with greater than 50% SOM, and none had greater than 69% (Table 2).

### Conversion Equations and OC:SOM Differences

The linear conversion equation derived from the primary mangrove dataset was:

OC = 0.511 (± 0.003) × SOM – 2.497 (± 0.174) (Supplementary Fig. S1) (Eq. 1).

A polynomial fit provided similar explanatory power, but with an intercept term that was closer to 0:

OC = 0.001 (± 0.000) × SOM^2^ + 0.380 (± 0.013) × SOM − 0.425 (± 0.262) (Supplementary Fig. S1) (Eq. 2).

Conversion equation slopes of the 17 study regions ranged from a low of 0.240 in Marapanim (MP), a delta setting in northern Brazil, to a high of 0.687 for the terrigenous lagoon samples in Laguna Gandoca, Costa Rica (Table 3).

**Fig. 3:**
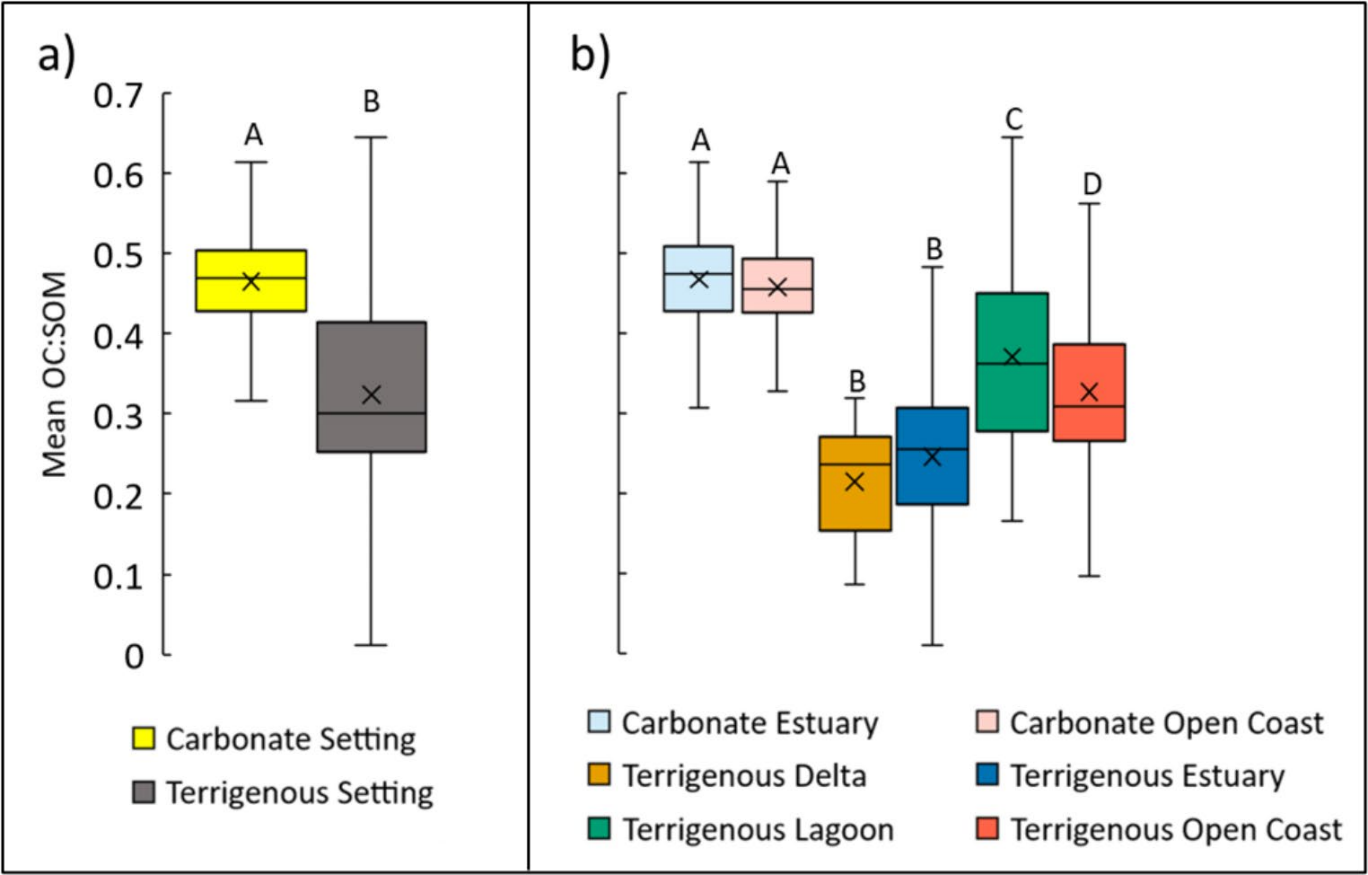
Boxplots of OC:SOM for all soil core intervals grouped by **(a)** sedimentary setting, and **(b)** coastal environmental setting. Boxes and whiskers represent median and interquartile ranges; X figures within boxes represent means. Different capital letters indicate significantly different means within each panel (p < 0.05)

The OC:SOM of samples from carbonate settings (0.465 ± 0.003) were greater than samples from terrigenous settings (0.323 ± 0.007) (t_(1244)_ = 24.103, p < 0.001) (Fig. 3a). Conversion equations for the two sedimentary settings were best represented by quadratic forms (Table 3). The terrigenous setting equation had almost twice the concavity of the carbonate setting equation (Supplementary Fig. S2a). The greater concavity of the terrigenous setting, with increasing slope steepness at high SOM, is due to the unexpectedly high OC:SOM of the Laguna Gandoca region. This region, which provided all the data for our terrigenous lagoon class, will be discussed further below.

The conversion equations specific to each coastal environmental setting were separated into two groups according to their similar slopes (Table 3; Supplementary Fig. S2b). The carbonate estuary, carbonate open coast, and terrigenous lagoon settings had slopes ranging from 0.470 to 0.687. Slopes for the terrigenous delta, estuary, and open coast settings ranged from 0.250 to 0.287 (Table 3). The terrigenous lagoon data are all from the Laguna Gandoca region. As a result, the terrigenous lagoon conversion slope looks very similar to the slopes of the two carbonate settings. Carbonate estuaries and open coasts had the highest OC:SOM (0.467 ± 0.003 and 0.458 ± 0.005, respectively), followed by terrigenous lagoons (0.370 ± 0.009), terrigenous open coasts (0.327 ± 0.014), and terrigenous deltas and terrigenous estuaries, which were not different from each other (0.215 ± 0.010 and 0.247 ± 0.17, respectively) (F_(5,1241)_ = 162.76, p < 0.001) (Fig. 3b).

**Fig. 4:**
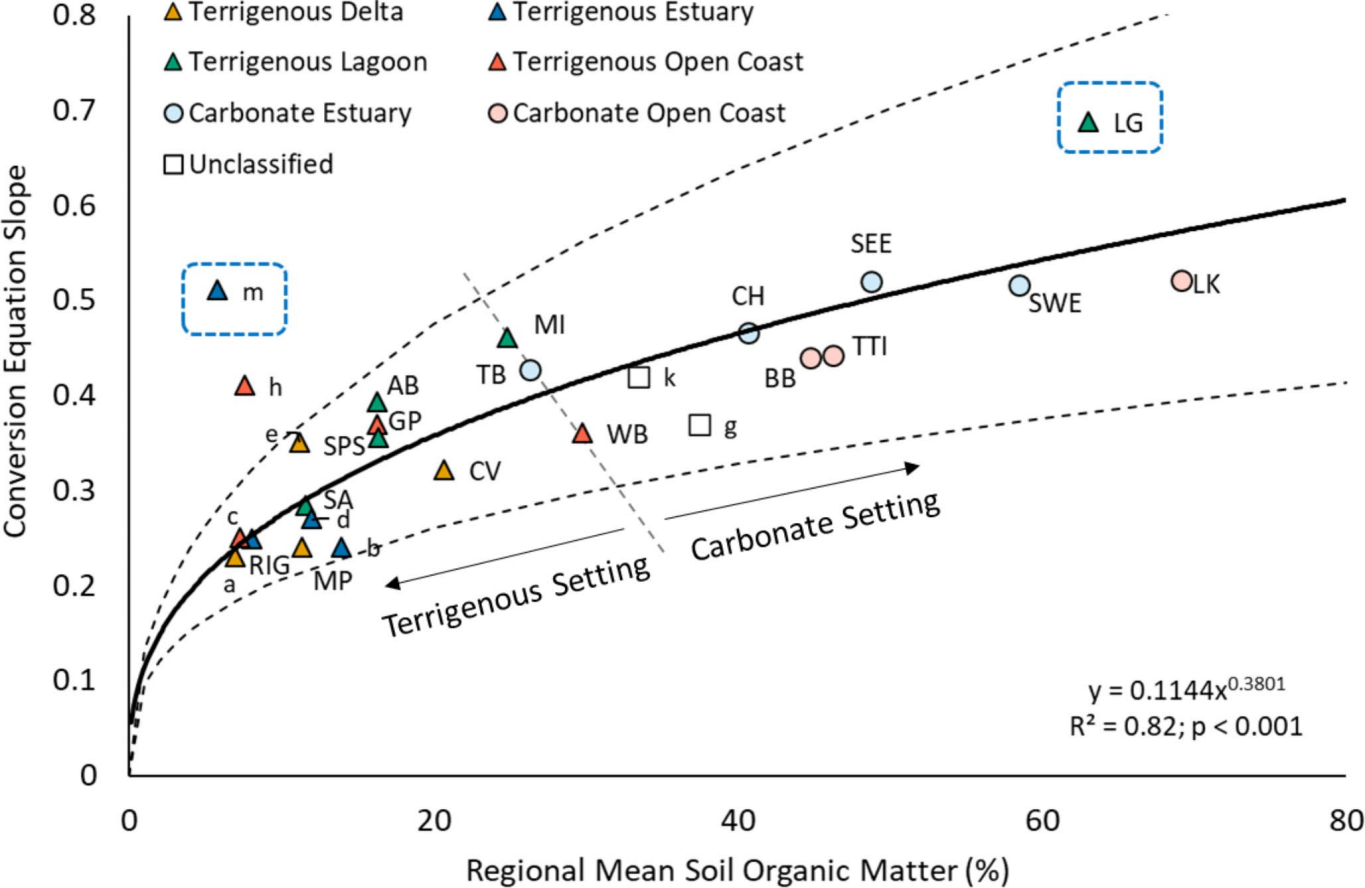
Regional conversion equation slopes from this dataset organized by coastal environmental setting and plotted as a function of regional mean soil organic matter content. Dashed black lines represent 1 SE of the best fit equation. Dashed blue boxes represent anomalous data points that are evaluated below. Note: literature values are plotted for context as lowercase letters but were not included for calculation of the best fit regression line. Lowercase letters refer to the references in Table 2. References included without designation of a coastal environmental setting are because the data used to derive the conversion equation was from multiple locations and settings. The following references from Table 2 were excluded from this comparison for the following reasons: Rovai et al. (2021a; Radabaugh et al. (2018) are already included in our primary dataset; Kauffmann and Donato (2012) and Gress et al. (2017) were excluded because no mean regional SOM value was provided; Chaikaew and Chavanich (2017) was excluded because OC was not directly measured but was calculated as total carbon minus inorganic carbon and thus represents a different method than used for calculation of these data; Delvecchia et al. (2014) was excluded as the authors noted poor precision of 2–45% for their OC measurements

**Fig. 5:**
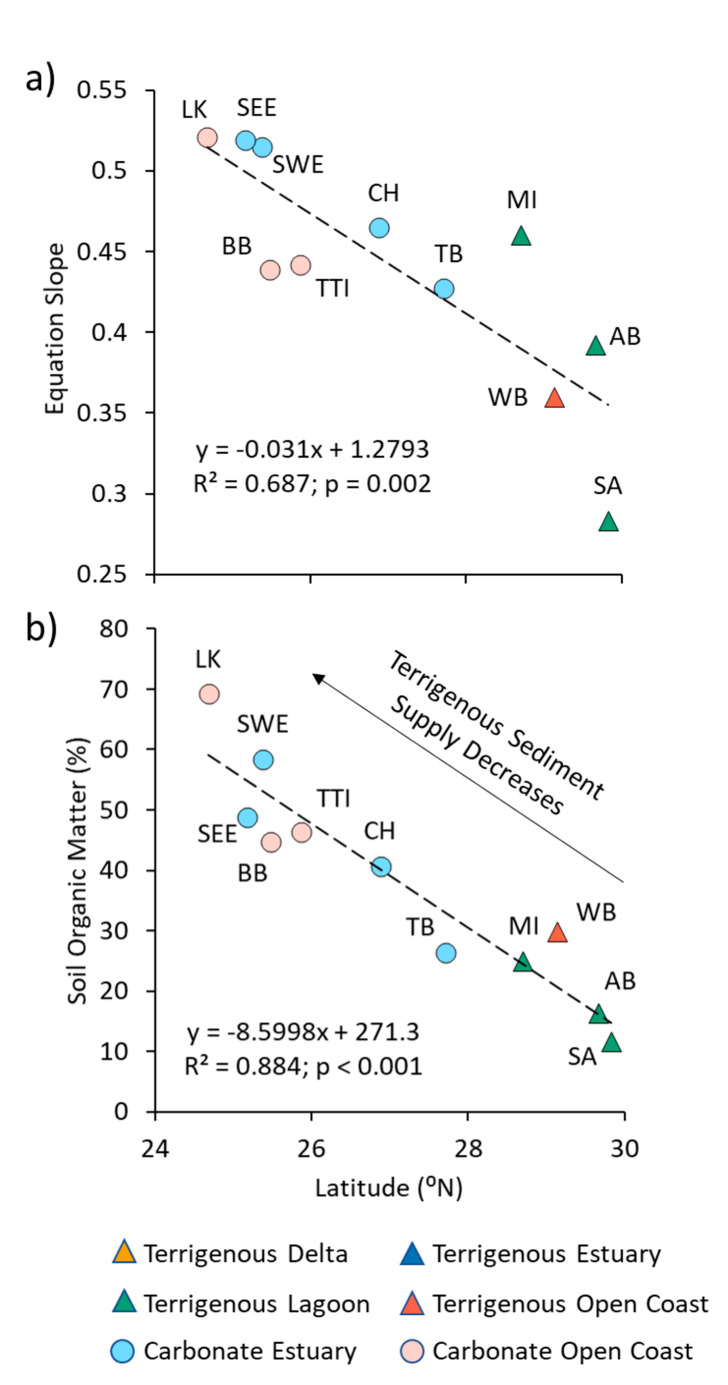
The geology of Florida occurs along a north-south orientation gradient, which leads to latitude predicting **(a)** the slope, and **(b)** mean soil organic matter content of these eleven regions. Latitude here represents a sedimentary gradient related to the increasing presence of carbonate bedrock and mud in south Florida. This latitudinal pattern was not discernible in the other regions of this study or the literature

Regional mean SOM content provided a strong prediction for the slopes of each regional conversion equation (Fig. 4; R^2^ = 0.82; p < 0.001). The power regression indicates an initial sharp rise when SOM is less than 10%, followed by a gradual flattening of the curve up to a maximum average slope of approximately 0.60 (Fig. 4). This regression identifies the dependence of conversion slopes on regional mean SOM content and indicates a general division between carbonate and terrigenous settings (Fig. 4). Terrigenous setting mangrove soils generally had less than 30% SOM and OC:SOM that ranged from 0.24 to 0.46 (although see below for more discussion of Laguna Gandoca which had unusually high values compared to other terrigenous setting regions). Carbonate setting mangrove soils generally had greater than 26% SOM and OC:SOM ranging from 0.43 to 0.52. The demarcation in this plot between carbonate and terrigenous regions occurs along a line composed of the Tampa Bay (TB), Waccasassa Bay (WB), and Merritt Island (MI) regions which are located along the transition from terrigenous dominant to carbonate dominant mineral sediment in central peninsular FL (Figs. 2 and 5). Results from previously published mangrove equations (Table 2) are generally in good agreement with the observed trend in our data (Fig. 4).

Two locations are identified for their apparently anomalous position in this figure (dashed blue boxes in Fig. 4). First, the Laguna Gandoca (LG) region in Costa Rica was classified as a terrigenous lagoon even though it had the highest mean SOM and highest regional slope, plotting well above the carbonate setting regions. Samples for this region were collected from a lagoon 4 km from the mouth of the Sixaola River. A survey of coastal sediments indicated the presence of carbonates in the nearshore marine environments near Punta Mona, 5 km to the northwest of the lagoon, but the sediments at the mouth of Laguna Gandoca have negligible carbonate content and almost 100% clay, silt, and very fine sand (Cortés et al. 1998). We speculate that when the lagoon was disconnected from the larger Sixaola River it gradually became more P-limited (indicated by mean ± 1 S.E. N:P ratios of 54.5 ± 5.6; Rovai et al. 2018). Under these conditions, the site developed highly organic peat soils from increased root production to forage for nutrients in addition to a decrease in inorganic sediment supply causing the soil to resemble P- and sediment-limited carbonate soils in south FL (sites SEE, SWE and LK in Fig. 4) where N:P values range from less than 20 in seaward sites to elevated values of 30–117 in inland, peat-dominated regions (Rovai et al. 2018; Breithaupt et al. 2019). Although the LG site has characteristics similar to a carbonate setting, the mineral sediment in this region is undoubtedly siliciclastic and not carbonate. The second anomalous location (point m in the dashed blue box in Fig. 4) has a conversion equation slope that is unexpectedly high for its regional mean SOM of 5.8% (Table 2). This could be due to that study’s use of a lower LOI temperature (375˚C) which would result in lower SOM compared to combustion at 550˚ C, and would have a higher than expected ratio of OC:SOM. A third point (h), identified as terrigenous open coast, also fell above the dashed line representing 1 SE of the trend, but we identified no anomalous reasons for its values and expect that this represents natural variability.

### Why is There so much Variability in Conversion Equation Slopes?

These results suggest the LOI process and comparisons of OC content in SOM can provide insights about ecosystem properties between regions defined by sedimentary and coastal environmental settings. Rather than assuming a relatively constant ratio of OC:SOM in the range of 0.40–0.50, this work highlights the importance of recognizing the wider variability that can occur. We propose that this variability reflects the macroscale ecosystem differences of climate, geomorphology, and hydrology that drive soil formation in each coastal environmental setting (Rovai et al. 2018). This dataset provides evidence of conversion equation slopes increasing from 0.24 up to 0.69 as a function of increasing bulk SOM content (Table 3; Fig. 4). We propose three explanations for this range: (1) differences in source organic matter, and/or (2) differences in post-depositional enrichment or depletion of OC relative to the total SOM pool, and (3) environmental gradients that contribute to differences in both source material and degradation processes.

#### Differences in Source Vegetation

Differences between general blue carbon ecosystem conversion equations (Table 1; Supplementary Fig. S1) could be due to their different vegetation source material and varying inherent recalcitrance (Kida and Fujitake 2020). In fact, the general concept of OM recalcitrance as a significant controlling variable of decay in soils/sediments has been known for many years (Hedges and Keil 1995; Schmidt et al. 2011; Bianchi et al. 2018). The steepness of our general mangrove equations (Eqs. 1, 2) suggest mangrove soils are enriched in OC relative to bulk SOM in saltmarsh and seagrass environments (Supplementary Fig. S1; Table 1). Similar distinctions between ecosystem types are not apparent at regional scales, however. The slope variability of our regional mangrove equations (Table 3) is nearly identical to variability reported for saltmarsh equations (0.22–0.52) (Craft et al. 1991). This broad and overlapping variability within and among blue carbon ecosystems suggests that vegetation source exerts a relatively minor influence on differences between conversion equations overall.

Our finding of a high general slope for the aggregate mangrove data is in direct contrast to the mangrove equation proposed by Ouyang and Lee (2020). Their findings indicate mangroves have the shallowest slope and lowest overall OC:SOM of blue carbon ecosystems (Table 1; Supplementary Fig. S1). Their equation indicates OC:SOM ranges from 0.25 for soil with 5% SOM to 0.36 for soil with 90% SOM (Table 1). The range of these ratios is substantially lower than many of our data points (Table 3), lower than previously published equations for both saltmarsh and seagrass ecosystems (Table 1; Supplementary Fig. S1), and lower than eight of the 14 linear slopes published in the mangrove literature over the past decade (Table 2 and references therein). A possible reason for the low overall slope is that only 22 of 1534 data points (1.4%) used to derive their equation have SOM greater than 60%, and thus may not capture the steeper slope representative of high SOM samples characteristic of carbonate settings (Table 1, Supplementary Fig. S1). This suggests that their dataset is derived largely from terrigenous setting studies since the OC:SOM values representative of their conversion equation are highly similar to the OC:SOM values of our terrigenous setting equations (Fig. 3b).

#### Post-Depositional Enrichment or Depletion of OC Relative to SOM

Comparisons of conversion equations suggest OC:SOM is influenced by environmental factors that affect post-depositional aging and degradation. These factors may include isolation and stabilization of OC due to physical protection or chemical interaction with the mineral sediment (Lützow et al. 2006; Rothman and Forney 2007; Marschner et al. 2008; Kida and Fujitake 2020), the level of biological activity in the soils, and climate factors including temperature and precipitation (Schmidt et al. 2011). The finding that lower slopes correspond to more degraded material is supported by Klingenfuß et al. (2014) who found the OC:SOM of humic sands (0.41) was substantially lower than the values for vascular plant and sphagnum peat soils (0.49 and 0.58, respectively). Craft et al. (1991) proposed that, compared to emergent wetland vegetation for which the OC:OM was 0.40, soils with high SOM content showed an increase in OC:SOM up to 0.65 through the “accumulation of [more] reduced organic materials”. This line of reasoning proposes why OC:OM values may become more enriched, but it does not explain how they might become more depleted compared to the source vegetation. A mechanism is needed to explain how depletion might occur, decreasing values of OC:SOM to as low as 0.24 (Table 3; Fig. 4). One such degradation mechanism might be related to the accumulation of microbial necromass that can decrease soil C:N ratios as soils are aged (Miltner et al. 2012; Liang et al. 2019).

#### Environmental Gradients

Data from the Florida regions in this dataset demonstrate the influence of environmental gradients on OC:SOM and conversion equation slopes. Latitude was negatively correlated with regional slopes (R^2^ = 0.64; p = 0.005; Fig. 5a) and OC:SOM increased from north to south, almost doubling from 0.28 in St. Augustine to 0.52 in the Lower Keys (Table 3). However, latitude separately predicted regional mean SOM (R^2^ = 0.88; p < 0.001; Fig. 5b), indicating that the correlation with slopes is at least partially due to cross correlation. A north-south environmental gradient in sediment type is the primary driver of this latitudinal trend. Much of Florida is situated atop a carbonate platform and the presence of siliceous sediment throughout the peninsula decreases from north to south because of riverine and longshore transport redistributing and weathering sediment from the Appalachian Mountains (Hine et al. 2009). As a result, the Florida study regions transition from dominance of terrigenous sediment in the north to carbonate sediment in the south (Figs. 1 and 5b). The northern regions have lower mean SOM content because they are “diluted” with a more abundant and regular input of mineral sediment. However, there is some suggestion in our data that geologic factors are not the exclusive reason for these differences. When comparisons of mean OC:SOM of the Florida regions were isolated in 10% SOM increments to remove the confounding factor of regional differences in mineral sediment content, there were indications of an additional latitudinal effect (Supplementary Fig. S3). The average slope of the four significant trends is -0.04; this average increased slightly to -0.03 if the four non-significant slopes were included. This means that for every one-degree increase in latitude, the mean OC content of SOM decreased by 4% points. We can only speculate that the reason for this difference may be due to greater physical protection or chemical interaction with the siliciclastic mineral sediment in the northern sites (Lutzow et al. 2006; Rothman and Forney 2007; Marschner et al. 2008; Kida and Fujitake 2020), or it may be driven by the effect of temperature differences on SOM decomposition (Lützow and Kögel-Knabner 2009; Liu et al. 2017), particularly N mineralization as it is the second greatest average mass contributor after C to SOM. These initial findings suggest that poleward climate warming might have an effect on SOM retention and composition. These latitudinal trends were not discernible in our aggregate dataset for either SOM (R^2^ = 0.001) or conversion equation slope (R^2^ = 0.001).

It is also possible that gradients in source vegetation could influence differences in OC:SOM across broad spatial scales. For example, perhaps OC:OM in the vegetation of younger, less developed mangrove communities could be different from ratios in older, more mature communities, such as may occur spanning climate gradients where mangrove range expansion is occurring (Osland et al. 2022).

### Recommendations for Using and/or Constructing a Conversion Equation

A primary objective of this research was to improve the accuracy of future blue carbon stock and burial rate estimates for academic researchers and conservation or restoration practitioners. Below, we provide a hierarchical framework for determining the most appropriate conversion equation for using LOI and SOM to estimate OC, listed in order of decreasing accuracy.

#### Direct Measurement

Although it may be obvious, the most accurate approach is to measure OC directly rather than estimating it. Even with a strong R^2^ of 0.95 for the aggregate dataset (Eqs. 1, 2; Supplementary Fig. S1), the spread in OC values around the trend generally exceeded 10 percentage points over the range of SOM content, indicating there will always be uncertainties when a conversion equation is used. However, there are circumstances when direct measurements are not practical or possible, and using LOI to estimate OC is economical or necessary; such circumstances were the impetus for this investigation.

#### Create a Region-Specific Conversion Equation

If unable to directly measure OC for all samples, the next best results will be obtained from a conversion equation derived from a subset of samples for the region where the work is being conducted. Not surprisingly, the region-specific equations were the best at predicting each region’s OC content in our comparisons. The central tendencies for the residuals of 16 of the 17 regions were not statistically different from zero (p > 0.05; Supplementary Table S2). For the Ten Thousand Islands (TTI), the lone region where residuals were different from zero, the regional conversion equation underestimated OC by only 0.90% (Supplementary Table S2). When creating a region-specific equation, we recommend using a uniform distribution of samples with low, medium, and high LOI values within the region to best account for the influence of SOM content on conversion slopes. The spatial extent of a “region” is something that will need to be determined by individual users, taking care to ensure samples are taken from geologically and ecologically similar settings. As Table 2 documents, generating a regional equation with a strong correlation coefficient is not always possible. Our SOM results were derived by combustion at 550℃ for three hours, however we recognize that there are preferences for similar but different temperatures and durations. The results of our study represent findings from a consistent methodology applied in multiple coastal environmental settings; they are not an evaluation of how different temperature and duration regimes affect results. Rather than recommending one particular method, we suggest users should follow methods that are consistent with previous studies in similar settings to avoid generating equations that are biased by methods differences.

#### Use a Conversion Equation Specific to a Coastal Environmental Setting

When it is not possible to create a region-specific equation, we find that the best alternative is to use one of the six coastal environmental setting equations (Table 3). Practitioners should identify the coastal environmental setting of their study region by reviewing the classification scheme described by Worthington et al. (2020) and accessing the global biophysical mangrove typology via the Ocean Data Viewer at https://data.unep-wcmc.org/, keeping in mind that regionally specific expertise may supersede the global-scale typology. Residuals for these conversion equations were not different from zero for seven of the 17 regions (Supplementary Table S2). Additionally, the sum of the absolute value of the residuals (AbsSum) was the lowest of the non-regional conversion equations at only 14.43, and the mean of the absolute value of the residuals (AbsMean) was only 0.85, or less than 1% point (Supplementary Table S2). The region with the largest central tendency value for residuals when using the coastal environmental setting conversion equations was an underestimation of the OC content of the Lower Keys (LK) by 2.30% (Supplementary Table S2).

#### Use a Conversion Equation Specific to a Sedimentary Setting

If there is uncertainty about the coastal environmental setting to which a region should be assigned, using one of the two quadratic equations developed for sedimentary setting (Table 3) would be similarly useful. Regional residuals of these sedimentary setting models were not different from zero for four of the 17 regions (Supplementary Table S2). The AbsSum and AbsMean values of 17.15 and 1.01 respectively, were only slightly higher than the values for the coastal environmental setting equations. The greatest deviances were an overestimation of OC content by 2.06% points in the Caravelas (CV) region and an underestimation of the OC content by 2.42% points in the Merritt Island (MI) region. Users can identify which of the two equations is best suited for their samples by empirically checking samples for carbonate content using the secondary LOI process of combusting samples at 990℃ (Dean 1974; Breithaupt et al. 2019).

#### Use Linear or Quadratic General Mangrove Conversion Equations

The high correlation coefficients of the linear and quadratic general conversion equations derived from the aggregate dataset (Eqs. 1 & 2; Supplementary Fig. S1), indicate they provide a good general approach for mangrove soils. However, in comparison to the equations tailored to environmental characteristics, these aggregate dataset conversions showed some weaknesses at the regional level. Regional residuals were not different from zero for five and seven of the 17 regions, for the quadratic and linear models respectively (Supplementary Table S2). However, the AbsSum and AbsMean values were greater than for the coastal environmental setting and sedimentary setting models (Supplementary Table S2), but still relatively modest except for a few regions. For example the linear equation of the aggregate dataset overestimated OC by 3.56 and 4.81% points for the Caravelas (CV) and Waccasassa Bay (WB) regions, respectively. The aggregate quadratic conversion equation also overestimated OC at Caravelas and Waccasassa Bay by 3.43 and 3.95% points respectively, and underestimated OC in Charlotte Harbor (CH) and the Lower Keys (LK) by 3.08 and 3.48, respectively. These deviations resulted in the largest AbsMean value of 1.54 of the five conversion approaches derived from our primary dataset (Supplementary Table S2).

The existing global-scale mangrove conversion equation (Ouyang and Lee 2020) consistently under-estimated the carbon content of most soils in our 17 regions (Supplementary Fig. S1, Supplementary Table S2) likely because the dataset from which it was derived had few samples with high SOM content (Table 1). Overall, the AbsMean of the residuals for that Eq. (3.3%, Supplementary Table S2) was higher than any of the conversion approaches derived from our data, indicating an overall underestimation of OC across these regions by a total of 48.9% points (Supplementary Table S2). Two of our regions where the model performed well were Ratones, Itapoá, and Guaratuba (RIG), terrigenous estuaries, and the terrigenous lagoon sites in São Paulo State (SPS), both with average SOM content less than 20%.

Lastly, the power regression in Fig. 4 could be used to estimate a slope based on regional mean SOM. This equation assumes an intercept of zero for each regional slope, and our empirical results show this is rarely the case (Table 3) because of methodological uncertainties related to removal of both carbonates and water. Despite these limitations, the power regression (Fig. 4) provides a rough guide for an appropriate slope in the absence of other data.

### Global Implications

**Fig. 6:**
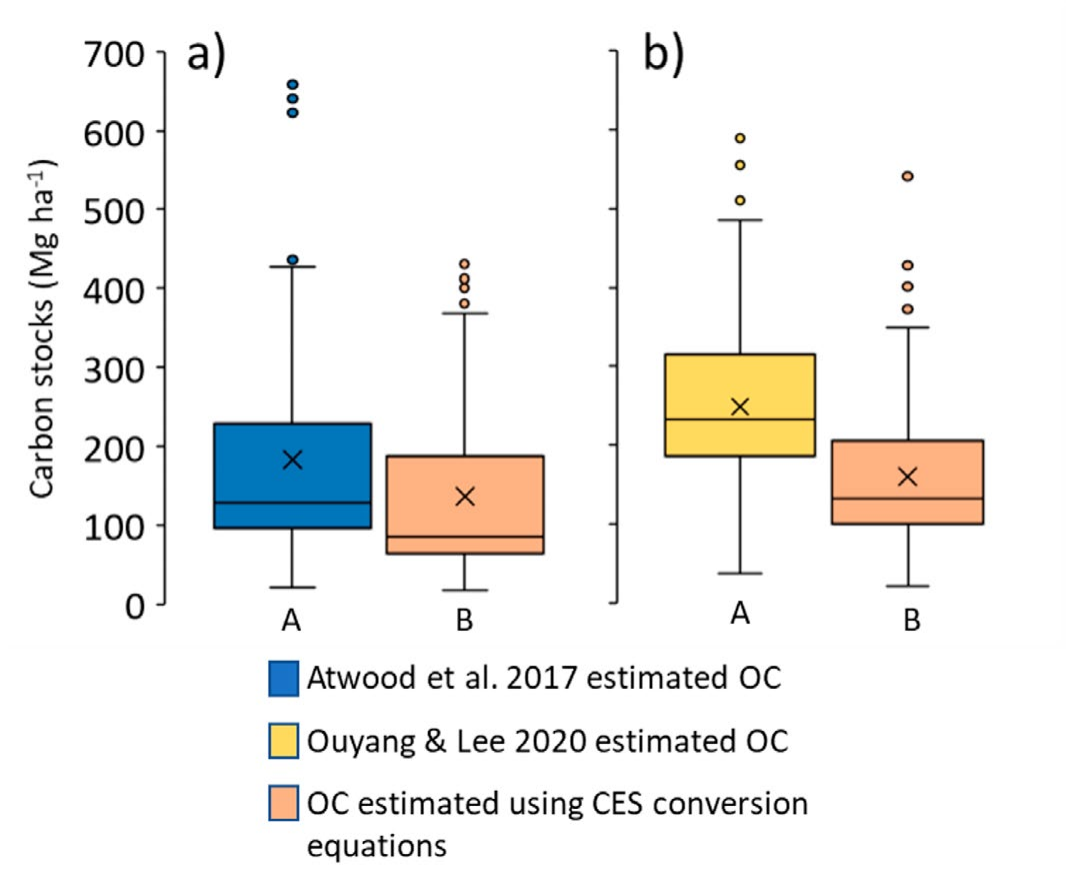
Comparison of estimated organic carbon (OC) stocks from the **(a)** Atwood et al. (2017), and **(b)** Ouyang and Lee (2020) datasets. Atwood et al. (2017) estimated OC by dividing LOI by 2.07. Ouyang and Lee (2020) estimated OC as 0.21×SOM^1.12^. Our estimates are derived using the coastal environmental setting (CES) equations from Table 3. Boxes and whiskers represent median and interquartile ranges; X figures within boxes represent means. Different capital letters beneath each box indicate significantly different medians within each panel (p < 0.05). Stock depths were specified as 1 m deep for Atwood et al. (2017), and multiple stock depths were aggregated by Ouyang and Lee (2020)

Assessing the global importance of this framework for achieving more accurate estimates of soil OC is difficult because global soil stocks, usually up to 1 m depth, are largely derived from direct measurements of OC. For two recent global mangrove soil OC stock assessments (Atwood et al. 2017; Ouyang and Lee 2020) 15% (132 of 872 samples) and 7% (112 of 1534 samples) were estimated from LOI, respectively. Note, for the Atwood et al. (2017) dataset we were unable to determine if OC was measured or estimated from an additional 201 samples from unpublished sources. It is less straightforward to quantify the frequency with which conversion equations are applied in the gray literature such as applied regional blue carbon conservation projects. Nonetheless, we found that use of the coastal environmental setting equations produced a significantly lower median estimate of soil stocks than the estimation methods used in the previous studies (Fig. 6a & b). Using the coastal environmental setting conversion equations, our median (and interquartile range) of 85.4 (64.3; 188.3) Mg ha^− 1^ was lower than the median of 128.0 (95.7; 229.4) Mg ha^− 1^ estimated by Ouyang and Lee (2020) for the 112 samples in their dataset that were converted to OC from LOI. Similarly, our median of 133.3 (99.8; 206.9) Mg ha^− 1^ was less than the median of 232.2 (187.1; 315.1) Mg ha^− 1^ estimated from 132 samples in the Atwood et al. (2017) dataset. The OC:SOM of those estimates are 33–43% greater than ours for terrigenous settings, and between 3% to -12% different for carbonate settings. These differences indicate the potential importance of further examining mangrove soil OC stocks in the context of the coastal environmental setting framework (Worthington et al. 2020).

### Future Research Needs

This research has advanced a novel, global framework for understanding differences in ratios of OC:SOM in mangrove soils as a function of SOM content as well as sedimentary and coastal environmental settings. However, these findings also raise several questions. Exploration of these unknowns will contribute to a more robust understanding of the LOI process and its ability to predict soil OC content. It will also inform our understanding about SOM preservation and degradation processes in different settings, thereby providing more informed global estimates of blue carbon stocks and fluxes. We conclude by identifying the following research questions for future investigation globally:

- What is the variation in OC:OM of source vegetation among and within blue carbon ecosystems? Does it vary as much as soil does?
- How does particle age affect OC:SOM? Craft et al. (1991) have proposed that aging and degradation increase ratios of OC:OM following plant tissue death. More data are needed to understand when, how, and at what rate soil OC:OM values deviate from source vegetation.
- It is likely that some low OC:SOM values may be attributed to LOI’s over-estimation of SOM in clay-rich soils (Heiri et al. 2001), but it is not clear to what extent this is the case as there are few mangrove studies that have specifically examined LOI values relative to variations in soil clay content.
- As noted earlier, conversion equations derived from samples with high SOM content (i.e., greater than 60%) is lacking for saltmarsh and seagrass ecosystems. It is unclear if this is because those ecosystems lack samples with high SOM content or whether such measurements have not been reported.

## Electronic Supplementary Material

Below is the link to the electronic supplementary material.

Supplementary Material 1

Supplementary Material 2

Supplementary Material 3

Supplementary Material 4

Supplementary Material 5

Supplementary Material 6

Supplementary Material 7

Supplementary Material 8

## Data Availability

All data used for these analyses are available in Supplementary Dataset files 1, 2, and 3.
