# Supplementary figures and images for: An Improved Framework for Estimating Organic Carbon Content of Mangrove Soils Using loss-on-ignition and Coastal Environmental Setting
Source: Wetlands (Wilmington). 2023 Jun 22;43(6):57. doi: 10.1007/s13157-023-01698-z (PMC10287774; doi:10.1007/s13157-023-01698-z)

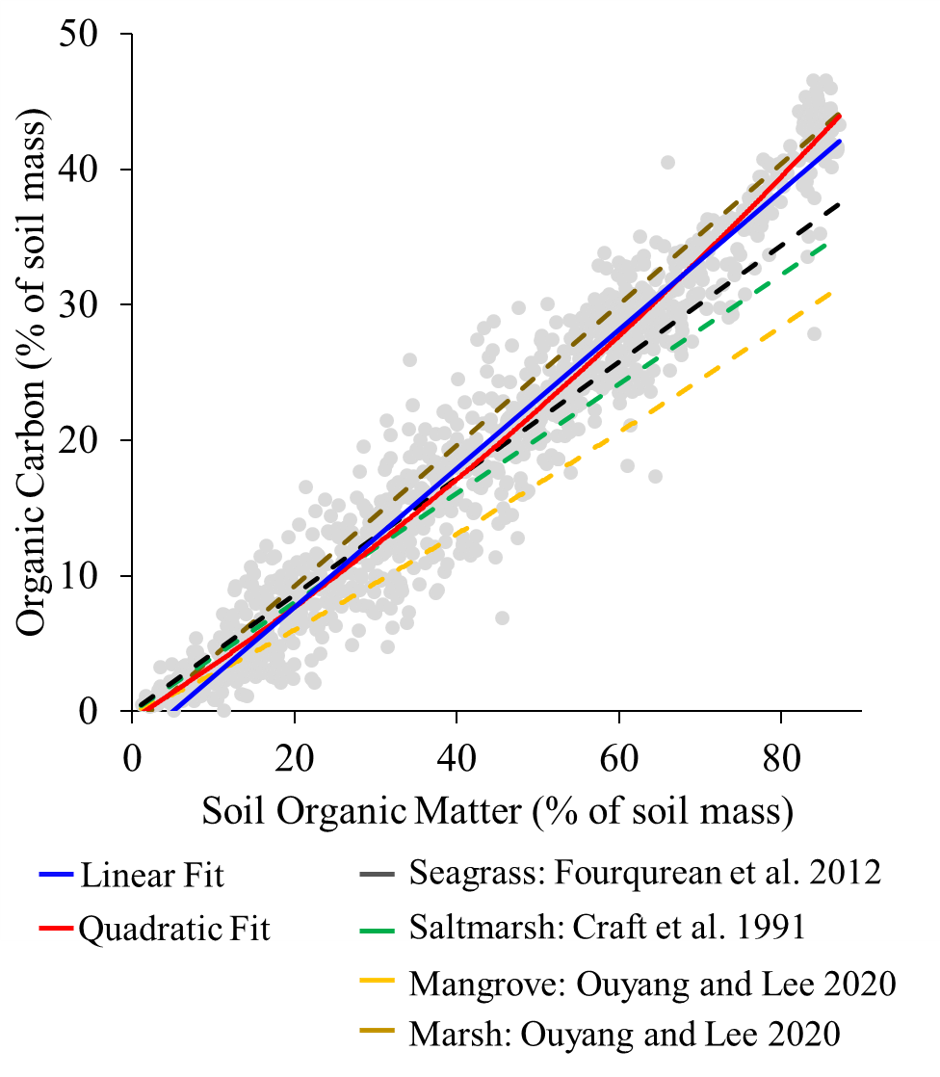

Supplement: Supplementary file 4 — Supplementary Material 4 [file 13157_2023_1698_MOESM4_ESM.tif]

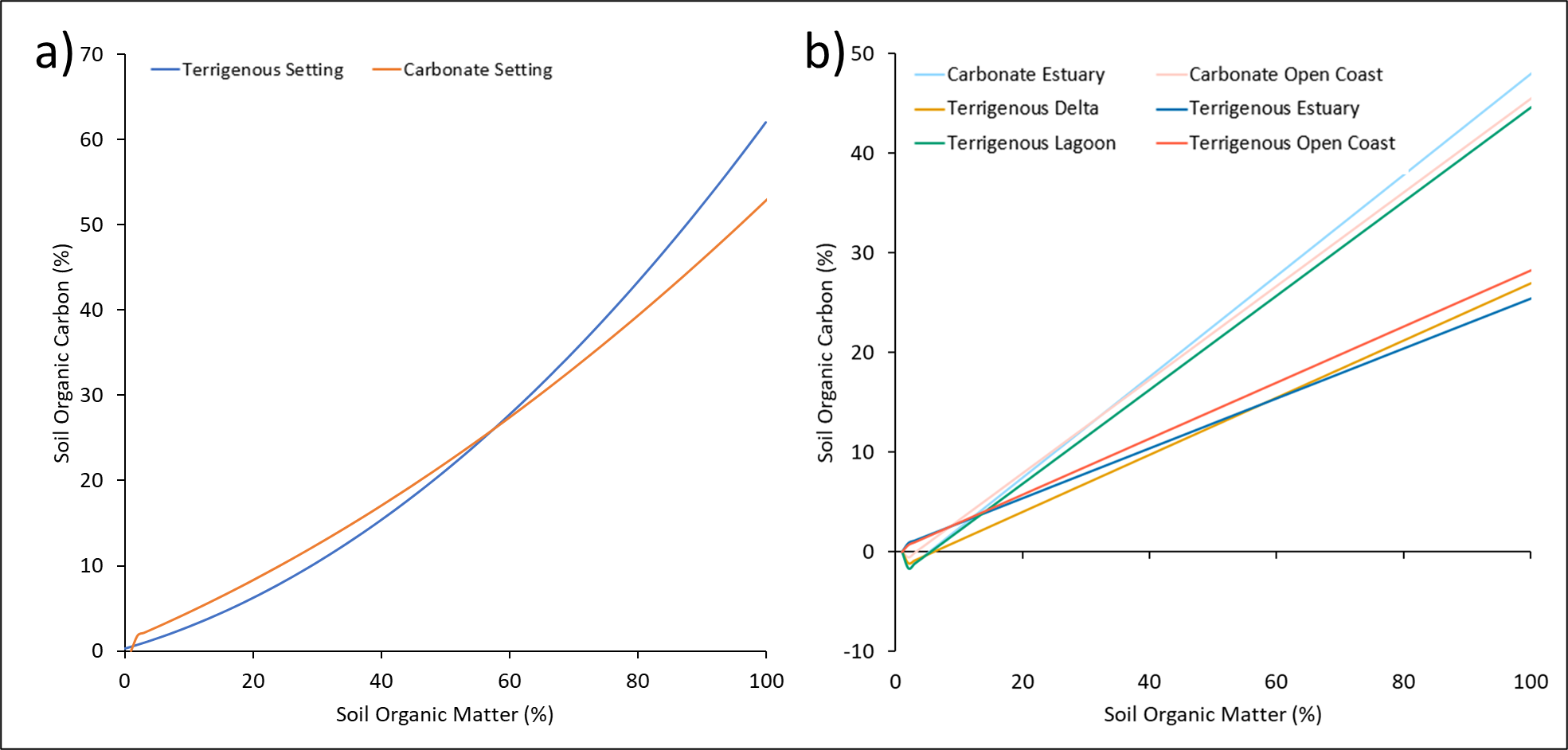

Supplement: Supplementary file 5 — Supplementary Material 5 [file 13157_2023_1698_MOESM5_ESM.tif]

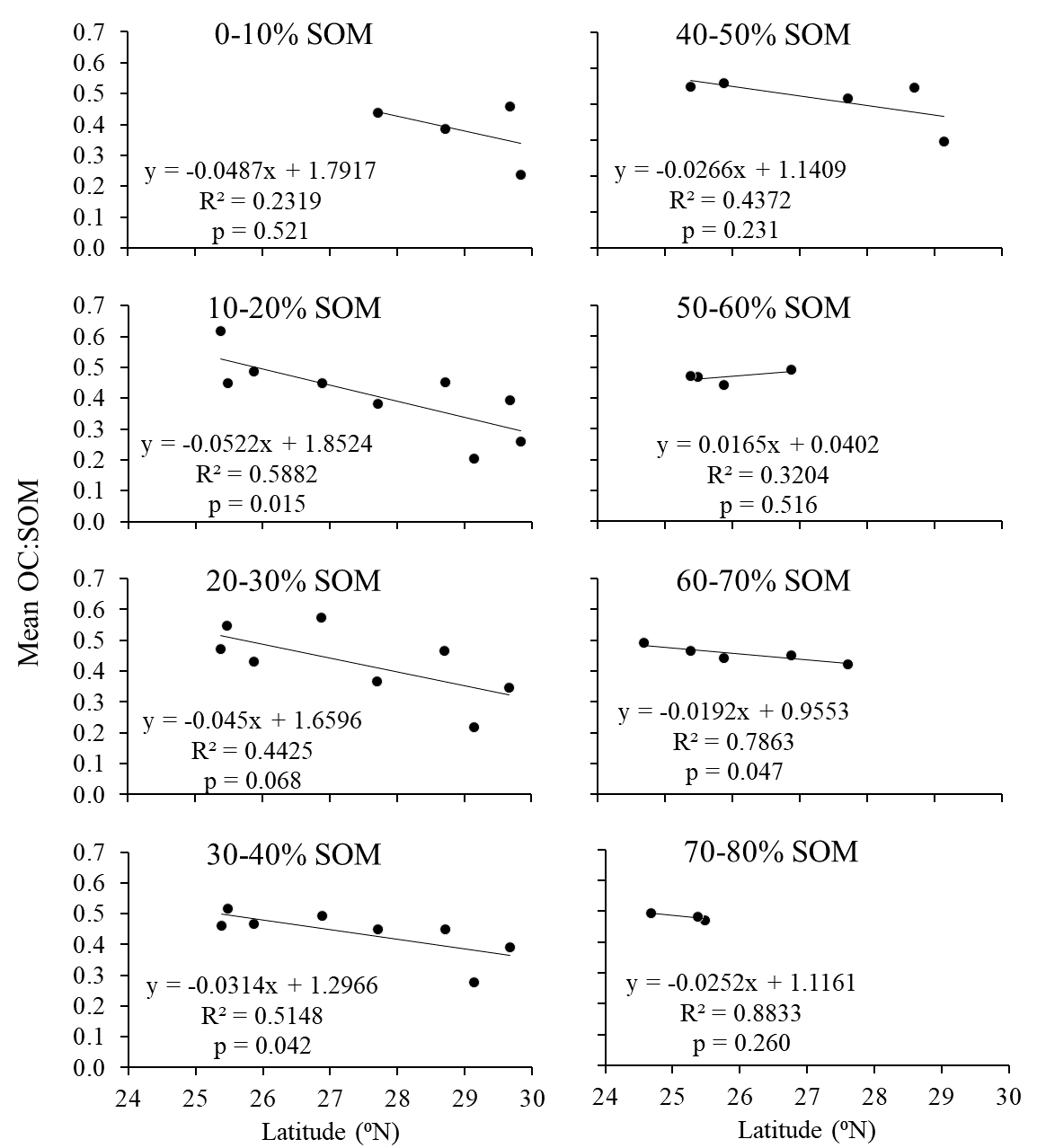

Supplement: Supplementary file 6 — Supplementary Material 6 [file 13157_2023_1698_MOESM6_ESM.tif]
